# Role of molecular functional imaging in present era of evidence based cancer medicine: to image or to imagine?

**DOI:** 10.1186/1470-7330-15-S1-P10

**Published:** 2015-10-02

**Authors:** A Mahajan, G Cook, V Goh, S Basu, M Thakur, A Weeks

**Affiliations:** 1 Tata Memorial Centre, Mumbai, India; 2 St Thomas’ Hospital, Kings College London, London, WC2R 2LS, UK

## Learning objectives

Molecular functional imaging (MFI) has given a newer insight to the medical imaging and has diversified the role of imaging in the field of the translational cancer medicine and has an indispensible role to play in screening, early diagnosis, staging, predicting prognosis, therapy delivery, therapy monitoring and follow-up. Overall there has been a significant development in the field of molecular imaging and its utilisation in the perspective of the biomedical research which has led to better understanding of the signalling pathways in the tumourigenesis and novel drug discoveries.

Review and Highlight the complementary role of these techniques in the detection and staging of tumours and which of these techniques is more adequate for clinical scenarios in oncology.

## Content organisation

• Cancer And Molecular Functional Imaging (MFI)

• MFI Of Gene Expression, Receptors And Signalling Pathways

• MFI of Multidrug-Resistance In Cancer

• MFI Of Extracellular Matrix And Its Key Components

• MFI Of Neoangiogenesis, Hypoxia And Metabolism

• MFI And Small Animal Imaging

## Conclusion

The future of molecular functional imaging in the coming era is its exploitation into understanding the gene expression profiling in-vivo and optimising the patient specific therapies using gene expression profiling. Quantitative molecular functional imaging, in conjunction with quantitative structural imaging, will be the future of 'personalised radiology,' 'personalised oncology,' 'personalised medicine,' and of oncologic research in the 21^st^ century and beyond.

